# Online-Sourced Retatrutide Complicating Impending Diabetic Ketoacidosis in a Patient With Type 1 Diabetes and Concurrent Shigella Gastroenteritis

**DOI:** 10.7759/cureus.115260

**Published:** 2026-08-26

**Authors:** Nassim Branine

**Affiliations:** 1 Acute Medicine, Edinburgh Royal Infirmary, Edinburgh, GBR

**Keywords:** diabetic ketoacidosis, gastroenteritis, glp-1 agonists, online medicines, peptides, research chemicals, retatrutide, shigella flexneri, type 1 diabetes mellitus, weight loss

## Abstract

Growing public interest in incretin-based therapies for weight loss has been accompanied by increasing availability of research peptides purchasable online. Products may be used without medical assessment or counselling regarding adverse effects, sick-day management or appropriate follow-up. In addition, composition, purity and dosing accuracy may be uncertain.

Retatrutide is a novel triple agonist of the glucose-dependent insulinotropic polypeptide, glucagon-like peptide-1 and glucagon receptors currently being investigated for obesity and type 2 diabetes mellitus, but has no established role in type 1 diabetes mellitus.

We report a man in his mid-30s with longstanding type 1 diabetes mellitus who presented with severe vomiting, diarrhoea, hyperglycaemia, ketonaemia and acute kidney injury shortly after self-administering an online-purchased product marketed as retatrutide for weight loss. His partner, who had taken the same preparation, also developed gastrointestinal symptoms. Both had also consumed a potential foodborne source of infection.

In type 1 diabetes mellitus, where patients remain dependent on exogenous insulin to suppress ketogenesis, gastrointestinal illness may rapidly precipitate ketosis when insulin is omitted and oral intake is reduced. Although diabetic ketoacidosis did not develop, peak ketonaemia reached 4.3 mmol/L in the setting of dehydration and insulin omission, requiring intravenous insulin and fluid replacement. During recovery, recurrent hypoglycaemia required intravenous dextrose and insulin dose adjustment, highlighting challenges of glycaemic management in acutely unwell patients exposed to agents with incretin and glucagon receptor activity.

Stool culture subsequently grew Shigella flexneri, introducing diagnostic uncertainty regarding the relative contributions of gastrointestinal infection and retatrutide exposure, and complicating counselling regarding future retatrutide use.

While causation cannot be established, the temporal relationship, similar symptoms in another exposed individual, and recognised gastrointestinal adverse-effect profile of retatrutide suggest that exposure may have contributed to symptom severity or amplified the metabolic consequences of infection. This case highlights diagnostic and management challenges created by unregulated research peptides obtained outside healthcare pathways, particularly in patients with type 1 diabetes mellitus where evidence to guide management is limited and clinicians in non-specialist settings may be unfamiliar with these agents. As access expands through online vendors and social media, clinicians should routinely enquire about prescribed and non-prescribed agents when assessing unexplained illness or metabolic decompensation.

## Introduction

Retatrutide is a novel triple agonist of the glucagon-like peptide-1 (GLP-1), glucose-dependent insulinotropic polypeptide (GIP), and glucagon receptors currently undergoing evaluation for obesity and type 2 diabetes mellitus. We report a case of a male in his mid 30s with longstanding type 1 diabetes mellitus who presented with severe vomiting, diarrhoea, hyperglycaemia, ketonaemia and acute kidney injury shortly after self-administering an online-purchased product marketed as retatrutide for weight loss. The product was not independently analysed or analytically verified to contain retatrutide, representing an important limitation when interpreting its potential contribution to the clinical presentation.

Although diabetic ketoacidosis did not develop, significant ketonaemia in the setting of dehydration and insulin omission necessitated intravenous insulin and fluid replacement. Stool culture subsequently grew Shigella flexneri, indicating a microbiologically confirmed contributor to the illness. 

While uncertainty remains regarding causation, this case highlights diagnostic and management challenges created by unregulated research peptides obtained online, particularly in type 1 diabetes mellitus where evidence to guide management is limited, and clinicians in non-specialist settings may be unfamiliar with these agents.

## Case presentation

A man in his mid 30s with a 17-year history of type 1 diabetes mellitus presented to the emergency department with persistent coffee-ground vomiting, loose watery diarrhoea and epigastric pain. He reported no dizziness or syncope before presentation. 

His diabetes was managed with basal-bolus insulin. His other medications included ramipril and tadalafil. His most recent HbA1c was 74 mmol/mol. 

Chronic diabetic complications included a remote history of diabetic ketoacidosis, elevated urinary albumin-creatinine ratio, background diabetic retinopathy with macular oedema, previous diabetic foot ulceration, and previous toe amputation. He was a non-smoker and had no recent alcohol use. He was 182cm tall with a weight of 95kg and a body mass index (BMI) of 28.7. 

Two days before admission, he self-administered a subcutaneous abdominal injection of an online-sourced product marketed as retatrutide for weight loss after viewing social media promoting its use and independently assessing the perceived risks and benefits. The product was supplied as a powder, labelled “not for human or veterinary consumption”, and was self-reconstituted with bacteriostatic water before being stored in the refrigerator. He reported intending to administer 0.5mg.

Approximately three hours later, he developed nausea, vomiting and diarrhoea that progressed over 48-72 hours. He reported multiple episodes of large-volume emesis and several episodes of dark brown coffee-ground vomit. Oral intake became minimal, and he omitted basal and bolus insulin the day before admission because of persistent vomiting and fear of hypoglycaemia. 

His partner administered the same preparation on the same day and also developed gastrointestinal symptoms, although these did not require medical intervention. Both individuals had also consumed improperly defrosted chicken soup approximately 48 hours before symptom onset. Epidemiologically, he had no contact with unwell individuals, no foreign travel, no large gatherings, no recent antibiotics, and no sexual contact with men. 

He reported no fever before or during admission. Examination demonstrated tachycardia and epigastric tenderness without guarding or peritonism. There was no haemodynamic instability.

Initial observations demonstrated sinus tachycardia (heart rate 117 beats/min), blood pressure 162/110 mmHg, respiratory rate 14 breaths/min, oxygen saturation 96% on room air, and temperature 37.1°C. Electrocardiography demonstrated sinus tachycardia. 

Venous blood gas analysis demonstrated preserved acid-base status (Table 1), indicating that diagnostic criteria for diabetic ketoacidosis were not met.

**Table 1:** Venous blood gas analysis on admission pCO₂: partial pressure of carbon dioxide.

Investigation	Result	Normal reference range
pH	7.381	7.35–7.45
Bicarbonate (mmol/L)	26.5	22–29
pCO₂ (kPa)	5.97	4.7–6.0
Lactate (mmol/L)	2.7	0.5–2.2

Initial laboratory investigations showed hyperglycaemia and acute kidney injury (creatinine 154 µmol/L; baseline 117 µmol/L). Haemoglobin was 134 g/L on admission and remained stable at 123 g/L on discharge despite episodes of coffee-ground emesis (Table 2).

**Table 2:** Laboratory investigations on admission, day of discharge, and one week post-discharge. WCC: white cell count; eGFR: estimated glomerular filtration rate; CRP: C-reactive protein. A dash (–) indicates that the investigation was not performed or no result was available at the specified time point.

Investigation	On admission	Day of discharge	1 week post-discharge	Normal reference range
WCC (×10⁹/L)	10.5	5.5	–	4.0–11.0
Neutrophils (×10⁹/L)	9.4	3.3	–	2.0–7.5
Sodium (mmol/L)	140	136	139	135–145
Potassium (mmol/L)	4.3	4.4	4.6	3.5–5.0
Chloride (mmol/L)	101	104	99	98–107
Bicarbonate (mmol/L)	23	–	–	22–29
Urea (mmol/L)	17.3	7.2	5.9	2.5–7.8
Creatinine (µmol/L)	154	111	117	60–110
eGFR (mL/min/1.73 m²)	45	>60	>60	>60
Amylase (U/L)	73	–	–	30–110
CRP (mg/L)	93	10	–	<5
Glucose (mmol/L)	17.8	–	–	4.0–7.8

A stool specimen obtained during admission was negative for C. diff, Cryptosporidium and Giardia. Viral stool polymerase chain reaction (PCR) was also negative for Norovirus, Adenovirus and Rotavirus. Stool culture subsequently grew Shigella flexneri and was sent to the reference laboratory, although the microbiological result became available only after discharge. 

The presentation was most consistent with gastrointestinal illness following exposure to a product marketed as retatrutide, with ketosis secondary to insulin omission in the context of type 1 diabetes mellitus, alongside subsequently confirmed Shigella flexneri infection. These processes may have interacted to produce the presentation. Acute pancreatitis was considered because of abdominal pain and vomiting but was thought unlikely given the normal amylase concentration and absence of other supporting clinical features. Toxbase was consulted for treatment guidance, but no specific guidance for retatrutide exposure was available. 

The patient was treated with intravenous crystalloid fluid replacement for dehydration and acute kidney injury. Given the presence of hyperglycaemia, significant recurrent ketonaemia and a history of insulin omission, a variable-rate intravenous insulin infusion was commenced alongside continuation of basal insulin replacement with input from the diabetes specialist team. A diabetic ketoacidosis protocol was not initiated because diagnostic criteria for diabetic ketoacidosis were not met. 

Coffee-ground emesis was managed conservatively with intravenous proton pump inhibitor therapy and antiemetics. Gastroenterology review concluded that upper gastrointestinal bleeding was most likely secondary to vomiting-induced oesophagitis or a Mallory-Weiss lesion. Endoscopy was not required because haemoglobin remained stable, there was no haemodynamic compromise, and symptoms improved with supportive treatment. 

Recovery was complicated by recurrent hypoglycaemia, which was attributed to reduced nutritional intake and was managed with intravenous dextrose administration and adjustment of insulin dosing. Glucagon rescue therapy was not required. 

Vomiting and diarrhoea resolved with improving oral intake, allowing discontinuation of variable-rate intravenous insulin and transition to his usual basal-bolus regimen. Renal function returned to baseline following intravenous fluid replacement and ketone concentrations normalised. No further episodes of coffee-ground emesis occurred. 

Before discharge, the patient was advised against further use of retatrutide and received education regarding carbohydrate counting, insulin adjustment and sick-day management. 

He was discharged after five days with a one-week course of omeprazole and arrangements for repeat renal function testing and diabetes outpatient follow-up. Ramipril was restarted following recovery of renal function. Although prescribed, omeprazole and antiemetics were not supplied on discharge and were obtained from his general practitioner the following day. 

After discharge, stool culture obtained during admission grew Shigella flexneri. As symptoms had resolved by the time the result became available, no further treatment was required. 

At follow-up, the patient reported complete resolution of gastrointestinal symptoms with no recurrence of ketosis or further hospital admissions. His diabetes management remained unchanged. He had not used retatrutide since but stated that, following confirmation of Shigella flexneri, he would consider using weight-loss injections again because he remained uncertain whether retatrutide had materially contributed to the illness.

## Discussion

Retatrutide (LY3437943) is a novel triple agonist of the glucagon-like peptide-1 (GLP-1), glucose-dependent insulinotropic polypeptide (GIP), and glucagon receptors. Unlike currently licensed GLP-1 receptor agonists, it combines incretin-mediated glucose lowering and appetite suppression with glucagon receptor-mediated increases in energy expenditure, representing a new generation of anti-obesity and glucose-lowering therapies [1-3]. The phase 3 efficacy and safety of retatrutide, a GIP, GLP-1, and glucagon receptor agonist, in people with type 2 diabetes and inadequate glycaemic control with diet and exercise (TRANSCEND-T2D-1) trial demonstrated significant reductions in HbA1c and body weight in adults with type 2 diabetes mellitus, with gastrointestinal adverse effects among the most frequently reported treatment-emergent adverse events [3].

Although retatrutide is being developed for obesity and type 2 diabetes mellitus, it has no established role in type 1 diabetes mellitus [1-3]. Limited observational data suggest tirzepatide and other incretin-based therapies improve glycaemic outcomes, body weight and insulin requirements in selected adults with type 1 diabetes mellitus, but no trials have been published for retatrutide [4-6]. Patients with type 1 diabetes mellitus remain dependent on exogenous insulin to suppress ketogenesis, including during periods of reduced food intake. Therapies associated with nausea, vomiting, delayed gastric emptying or appetite suppression may therefore increase vulnerability to ketosis when insulin doses are omitted [4,5]. In this case, severe gastrointestinal symptoms resulted in reduced oral intake, missed insulin doses, marked ketonaemia and acute kidney injury, although diabetic ketoacidosis did not develop [7].

An important aspect of this case was the subsequent identification of Shigella flexneri from stool culture obtained during admission. This provided a microbiologically confirmed contributor to the gastrointestinal illness and means that causation cannot be attributed solely to retatrutide exposure. The shared exposure to improperly defrosted chicken soup also supported a possible foodborne infectious source. However, the close temporal relationship between retatrutide administration and symptom onset, the occurrence of gastrointestinal symptoms in another individual who received the same preparation, and the recognised gastrointestinal adverse-effect profile of retatrutide suggest that the exposure may have contributed to symptom severity or amplified the metabolic consequences of the illness [1-3,8,9]. The temporal association is compatible with a drug effect, but the concurrent Shigella infection and unverified composition of the online-sourced product mean that causality cannot be established.

A synergistic interaction between retatrutide and Shigella infection also cannot be discounted. The absence of fever and inflammatory diarrhoea was atypical for shigellosis [10], raising the possibility that retatrutide altered the clinical expression of infection. GLP-1 receptor agonists delay gastric emptying and alter gastrointestinal motility, and emerging observational data suggest a possible association with small intestinal bacterial overgrowth [11], although whether this influenced disease severity remains speculative. This case illustrates how the adverse effects of novel triple agonists may complicate the diagnosis of concomitant gastrointestinal infection.

Regardless of whether retatrutide was the primary cause of the gastrointestinal illness, recent exposure significantly complicated inpatient management.

Recurrent hypoglycaemia developed during recovery, but glucagon rescue therapy was not used because clinicians were uncertain whether recent exposure to a glucagon receptor agonist might influence responsiveness to exogenous glucagon.

Experimental studies demonstrate glucagon receptor desensitisation with sustained stimulation [12]. Retatrutide has been engineered with lower glucagon receptor binding affinity than native glucagon to minimise this and has not demonstrated loss of efficacy in clinical trials [1], but the effects of recent exposure to a glucagon receptor agonist on the response to emergency glucagon administration have not been studied. Repeated glucagon receptor activation promotes hepatic glycogenolysis and gluconeogenesis, while prolonged fasting and reduced oral intake may deplete hepatic glycogen stores [13], providing a theoretically plausible physiological basis for this concern. In an absolute insulin-deficient state such as type 1 diabetes mellitus, these mechanisms in combination raise the possibility that glucagon rescue therapy might theoretically be less effective in some patients following severe gastrointestinal illness and recent exposure to a glucagon receptor agonist.

However, there is no clinical evidence that recent retatrutide exposure alters the effectiveness of emergency glucagon. In the absence of evidence or guideline recommendations, hypoglycaemia should continue to be managed according to established protocols.

This case highlights the challenges posed by online procurement of products marketed as "research chemicals". As the product's composition, concentration and purity were unverified, adverse effects may have reflected inaccurate dosing, contaminants, degradation products or substitution with another compound rather than the intended active ingredient [14,15]. Although labelled "not for human or veterinary consumption", it was marketed for weight loss without the medical assessment, counselling or monitoring that would normally accompany prescribing [14-16].

Although aware that retatrutide was unlicensed, widespread discussion on social media led him to perceive it as sufficiently established for personal use, suggesting that awareness of investigational therapies may outpace understanding of their regulatory status. He also reported feeling stigmatised after disclosure, which may discourage future reporting of non-prescribed therapies.

As retatrutide and other emerging metabolic therapies gain visibility online, clinicians are likely to encounter more patients sourcing these compounds outside regulated care. When assessing unexplained gastrointestinal symptoms, ketosis, or acute kidney injury, clinicians should routinely ask about prescribed and non-prescribed peptide use.

This case illustrates the diagnostic uncertainty and management challenges posed by medicines obtained outside standard clinical pathways, particularly where evidence is limited and clinicians may be unfamiliar with emerging therapies.

## Conclusions

This case highlights the diagnostic and management challenges associated with unregulated research chemicals, particularly emerging incretin-based therapies such as retatrutide. In a patient with type 1 diabetes mellitus, gastrointestinal symptoms and reduced oral intake following exposure to a product marketed as retatrutide were clinically important because insulin omission increased the risk of metabolic decompensation. The concurrent identification of Shigella flexneri demonstrated the need to consider infectious causes alongside medication-related adverse effects.

Clinicians should maintain a non-judgemental approach to enquiring about prescribed and non-prescribed therapies, including research chemicals obtained online. As retatrutide and other emerging therapies gain visibility through clinical research, online vendors and social media, awareness of their potential adverse effects and implications for patients with pre-existing disease will be increasingly important for recognising exposures that may otherwise complicate diagnosis and management.
